# Ultrastructural morphology is distinct among primary progenitor cell isolates from normal, inflamed, and cryopreserved equine hoof tissue and CD105^+^K14^+^ progenitor cells

**DOI:** 10.1007/s11626-019-00380-1

**Published:** 2019-07-11

**Authors:** Qingqiu Yang, Mandi J. Lopez

**Affiliations:** Laboratory for Equine and Comparative Orthopedic Research, Department of Veterinary Clinical Sciences, School of Veterinary Medicine, Louisiana State University, 1909 Skip Bertman Dr., Baton Rouge, LA 70803 USA

**Keywords:** Horse, Cryopreservation, Stem cell, Microscopy, Scaffold

## Abstract

The equine hoof dermal-epidermal interface requires progenitor cells with distinct characteristics. This study was designed to provide accurate ultrastructural depictions of progenitor cells isolated from inflamed tissue and normal tissue before and after cryopreservation and following selection of cells expressing both keratin (K) 14 (ectodermal) and cluster of differentiation (CD) 105 (mesodermal). Passage 3 cell ultrastructure was assessed following 2D culture and after 3D culture on decellularized hoof tissue scaffolds. Outcome measures included light, transmission electron, and scanning electron microscopy, immunocytochemistry, and CD105^+^K14^+^ cell trilineage plasticity. Cells from normal tissue had typical progenitor cell characteristics. Those from inflamed tissue had organelles and morphology consistent with catabolic activities including lysosomes, irregular rough endoplasmic reticulum, and fewer vacuoles and early endosomes than those from normal tissue. Cryopreserved tissue cells appeared apoptotic with an irregular cell membrane covered by cytoplasmic protrusions closely associated with endocytic and exocytic vesicles, chromatin aggregated on the nuclear envelop, abundant, poorly organized rough endoplasmic reticulum, and plentiful lysosomes. Cells that were CD105^+^K14^+^ were distinguishable from heterogenous cells by infrequent microvilli on the cell surface, sparse endosomes and vesicles, and desmosomes between cells. Cells expressed ectodermal (K15) and mesodermal (CD105) proteins in 2D and 3D cultures. Inflamed and cryopreserved tissue isolates attached poorly to tissue scaffold while normal tissue cells attached well, but only CD105^+^K14^+^ cells produced extracellular matrix after 4 d. The CD105^+^K14^+^ cells exhibited osteoblastic, adipocytic, and neurocytic differentiation. Ultrastructural information provided by this study contributes to understanding of equine hoof progenitor cells to predict their potential contributions to tissue maintenance, healing, and damage as well post-implantation behavior.

## Introduction

The equine hoof originates from a dermo-epidermal anlage to form what is considered one of the most complex mammalian integumentary structures (Bragulla and Hirschberg 2003). A branched, interdigitating interface between the epidermal and dermal layers, the lamellar stratum internum, suspends the bony third phalanx from the cornified hoof capsule (Pollitt 1998; Pollitt 1992). The interface both withstands the physiologic forces of ambulation and participates in hoof tissue homeostasis (Stark et al. 2004). The unique tissue architecture composed of redundant, dermal, and epidermal tissue extensions creates an expansive surface area with innumerable cell tight junctions within minimum volume. Inflammation of the stratum internum, laminitis, has severe consequences on the tissue’s structural integrity and can cause permanent disfigurement and compromised function (Johnson et al. 2000; Patterson-Kane et al. 2018).

Ultrastructural features are invaluable to understanding cell activities, interactions, and identity, as well as the consequences of pathology (Camussi et al. 2010; Kamakura et al. 2013). Ultrastructure of the stratum internum, including desmosome cell junctions and a basement membrane interface between epidermis and dermis, is described (Leach and Oliphant 1983; Pollitt 1994) as are pathologic changes in post-mortem samples from naturally occurring and artificially induced inflammation (French and Pollitt 2004; Nourian et al. 2009). However, viable, cultured hoof cell morphology is vitally important to understanding both normal and pathologic behavior and signaling. To date, ultrastructural evaluation of the cellular component of the lamellar tissue interface is largely unexplored (Linardi et al. 2015).

Knowledge about cells that occupy the unique dermal-epidermal niche are primarily limited to human skin and fingernail, and there is a dearth of information about those cells that reside in the same interface within the equine hoof. Transitional progenitor cells that share features of the transitional epithelium exist among basal cells at the squamous-columnar junction in the mouse upper gastrointestinal tract (Jiang et al. 2017). The cells are thought to play a central role in tissue maintenance, including the basement membrane of the dermal-epidermal junction (Stark et al. 2004). Comprehensive ultrastructural information is needed to fully elucidate the progenitor cell population of the equine hoof dermal-epidermal interface.

A notable consideration for contemporary progenitor cell biology is the challenges of distance and associated time between tissue harvest and processing sites. Cryogenic tissue and/or cell preservation are frequently required (Engelmann et al. 1994). Cryopreservation is known to alter the behavior and morphology of progenitor cell isolates (Duan et al. 2018), though surrounding tissues are thought to provide some protection to cells in vessel and bone tissue during stasis (Pascual et al. 2004; Bakhach 2009). Direct comparison of cell isolates from fresh and cryopreserved tissue helps predict morphologic changes manifest by cryogenic preservation.

Decellularized tissue templates that provide a natural tissue niche to instruct exogenous progenitor cells are popular for de novo tissue generation (Gilpin and Yang 2017; Urciuolo and De Coppi 2018). The quantity and organization of extracellular matrix produced by progenitor cells on tissue templates are often used to discern cell neotissue forming capabilities (Liu et al. 2013). Cell-scaffold interactions that drive matrix deposition, composition, and organization vary among cell morphologies as evidenced by unique responses among progenitor cells from different tissues to identical decellularized scaffold (Booth et al. 2012; Murphy et al. 2013; Wang et al. 2013a; Agmon and Christman 2016). Behaviors also vary between cells from normal and inflamed tissues (Tang et al. 2016). Additionally, a strong predictor of progenitor cell potential for functional tissue formation is preservation of characteristics in both 2D and 3D culture (Duval et al. 2017). As such, evaluation of hoof progenitor cell behavior on decellularized laminar tissue provides a natural foundation for de novo hoof tissue generation and anticipation of in vivo behavior (Agmon and Christman 2016).

Recent studies support the promise and potential of progenitor cells to contribute to and direct bone tissue formation in vitro and in vivo (Labibzadeh et al. 2016; Tracy et al. 2016). Differences among species, progenitor cell tissue sources, and freezing techniques preclude assumptions or predictions about progenitor cells without explicit evaluation (Elahi et al. 2016; Ren et al. 2009).

This series of investigations provides essential details about the in vitro ultrastructure of equine hoof progenitor cells from normal, inflamed, and cryopreserved tissue. The ultrastructure of a novel transitional progenitor cell immunophenotype is also presented. Potential for generation of 3D cell-tissue template constructs is explored by ultrastructural assessment of cultured progenitor cells of each description on decellularized stratum internum tissue templates. In all, the objective of the work is to provide novel and accurate depictions of progenitor cells isolated from the unique dermal-epidermal niche of equine hoof tissue exposed to distinct and meaningful conditions.

## Materials and Methods

### Tissue harvest

Cells were harvested immediately post-mortem from six horses (Table 1) with and without a history of chronic laminitis that were humanely euthanized for reasons unrelated to this study. Briefly, following aseptic preparation of the hoof surface, the stratum internum was exposed by elevation of the cornified hoof wall between two parallel, full thickness cuts (approximately 6 cm apart) extending from proximal to distal on the dorsal hoof. Two contiguous regions of stratum internum (2 × 2 cm) were sharply excised with a sterile no. 20 scalpel blade and, following a rinse with 0.01% chlorhexidine, maintained in phosphate-buffered saline (PBS, Hyclone™, Logan, UT), containing 1% penicillin at 4°C for approximately 1 h. One sample was then placed in 1 ml cryopreservation medium (10% dimethyl sulfoxide (DMSO), 10% Dulbecco’s modified Eagle’s medium with Ham’s F12 nutrient mixture (DMEM-Ham’s F12, Hyclone, Logan, UT), 80% fetal bovine serum (FBS, VWR Life Science, Radnor, PA)) within a microvial (Fisher Scientific, Roskilde, Denmark) and cooled to − 80°C at which they were maintained for 1 wk (CoolCell™ LX, BioCision, Tewksbury, MA).

**Table 1:** Sample outcome measures

Age (yr)	Condition	Sex	Breed	Actin stain	TEM	CD 105+, K14+ cell isolation	SEM
5	Unaffected, cryopreservation	Gelding	Thoroughbred	+	+	+,−	+
7	Unaffected, cryopreservation	Gelding	Thoroughbred	+	+	+,−	+
12	Unaffected, cryopreservation	Mare	Quarter horse	+	+	+,−	+
9	Laminitic, cryopreservation	Gelding	Thoroughbred	+,−	+,−	+,−	+,−
7	Laminitic, cryopreservation	Mare	Quarter horse	+,−	+,−	+,−	+,−
18	Laminitic, cryopreservation	Mare	Thoroughbred	+,−	+,−	+,−	+,−

*TEM* transmission electron microscopy, *SEM* scanning electron microscopy

### Cell isolation and culture

Cryopreserved tissue was thawed at room temperature for 5 min and washed three times with PBS to remove cryopreservation medium. Fresh and thawed tissue was diced into cubes (5 mm × 5 mm) and added to 50-ml sterile tubes containing 0.1% collagenase digest (0.1% bovine serum albumin (BSA, Sigma-Aldrich, St. Louis, MO), 0.1% collagenase type-1 (Worthington Biochemical Corporation, Lakewood, NJ) in DMEM-Ham’s F12 medium) at a ratio of 1:2 tissue to digest (*v*/*v*). Digests were maintained at 37°C for 2 h with three-dimensional agitation (5.5×*g*, GyroTwister™ GX-1000, Labnet, Inc., Edison, NJ). The mixture was passed through a 100-μm followed by a 70-μm filter (Fisher Scientific, Waltham, MA) and centrifuged at 260×*g* for 5 min. Cell pellets were suspended in stromal culture medium (10% FBS, 1% antibiotics in DMEM-Ham’s F12 medium), and cells were seeded on 10-mm tissue culture plates (Fisher Scientific, Denmark) at a density of 5 × 10^3^ cells/cm^2^. Medium was refreshed every 3 d, and cells were passaged at 80% confluence following trypsin (Hyclone, Logan, UT) detachment and hemocytometer quantification. Standard culture conditions were used (5% CO_2_, 37°C).

### CD105^+^K14^+^ cell isolation

**Figure 1.**
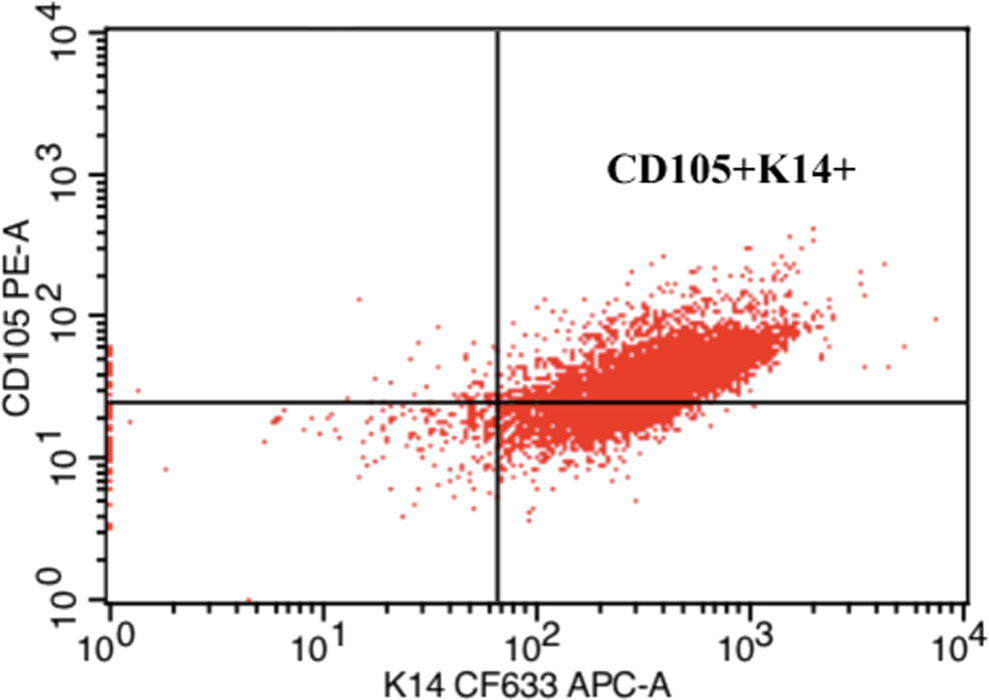
Representative scatter plot demonstrating fluorescence activated cell sorting gating technique used to separate CD105^+^K14^+^ cells from heterogenous primary cell isolates.

Cells from fresh tissue were incubated with polyclonal antibodies, labeled CD105-PE (Mouse, eBioscience no. 12-1057-42, San Diego, CA), and unlabeled K14 (Mouse, Fisher Scientific, no. MA5-11599, Rockford, IL) to which a dylight 633 label (Fisher Scientific) was added at a concentration of l μl (0.2 μg)/1 × 10^6^ cells in darkness for 40 min. Cells expressing both antibodies were selected with a FACSCalibur flow cytometer and Cell Quest Pro software (BD Biosciences, San Jose, CA) (Fig. 1).

### Cell cytoskeleton morphology

Cells were added (5 × 10^3^ cells/cm^2^) to six-well culture plates (Fisher Scientific, Denmark) and cultured in stromal medium for 7 d. Following three rinses with PBS, cells were fixed in 4% paraformaldehyde at 4°C overnight. Plates were rinsed with PBS, and cells were permeabilized with 1% Triton X-100 for 20 min at room temperature followed by incubation with Acti-stain™ 488 phalloidin (2 mg/ml, 1:150, no. PHDG1-A, Cytoskeleton Inc., Denver, CO) according to the manufacturer’s instructions. Nuclei were stained with Hoechst 33342 dye (10 mg/ml, 1:1000, no. H1399, Invitrogen, Carlsbad, CA), and results were viewed with a fluorescent microscope (DM5000B, Leica, Buffalo Grove, IL) fitted with a digital camera (DFC 480, Leica).

### CD105^+^K14^+^ cell multilineage differentiation (adipogenic, osteogenic, neurogenic)

Cells were cultured in six-well plates (Fisher Scientific, Denmark) with stromal medium until 80% confluence when the culture medium was changed to one of three induction media as described below.

### Adipogenesis

Cells were cultured in adipogenic induction medium (DMEM-Ham’s F12, 3% FBS, 1% antibiotic solution, biotin (33 mmol/L), pantothenate (17 mmol/L), insulin (1 mmol/L), dexamethasone (1 mmol/L), isobutylmethylxanthine (IBMX, 0.5 mmol/L), rosiglitazone (5 mmol/L) (TZD, AK Scientific, Union City, CA), 5% rabbit serum (Invitrogen Corporation, Carlsbad, CA)) for 3 d followed by adipogenic maintenance medium (adipogenic medium minus IBMX and rosiglitazone) for 2 d. The presence of intracellular lipids was confirmed by staining with oil red O for 20 min after cells were fixed overnight in 4% paraformaldehyde at room temperature and then washed with PBS. An inverted phase contrast microscope (Olympus® CKX41SF, Japan) instrumented with a digital camera (Olympus DP21, Japan) was used to obtain digital images.

### Osteogenesis

Osteogenic induction medium (DMEM-Ham’s F12, 10% FBS, 1% antibiotic solution, β-glycerophosphate (10 mmol/L), dexamethasone (20 nmol/L), sodium 2-phosphate ascorbate (50 mg/ml)) was used to culture cells for 14 d. They were then fixed with 70% cold ethanol overnight. Colonies were stained with 2% alizarin red in distilled water (pH 4.2) for 15 min at room temperature and then rinsed with distilled water to confirm the presence of calcium. Digital images were obtained as described above.

### Neurogenesis

For neurocytic induction, cells were cultured in neurogenic pre-induction medium (DMEM, 10% FBS, 1 mM 2-mercaptoethanol) for 2 d and then for 3 d in neurogenic induction medium (DMEM, 5.5 mM glucose, 120 μM indomethacin, 10% FBS, 3 μg/ml insulin, 300 μM IBMX) for 3 d. Paraformaldehyde (4%) was used to fix the cells overnight. Samples were then blocked with 10% goat serum for 1 h at 37°C. A mouse anti-Map 2 antibody (Mouse, 0.5 mg/ml, 1:100, no. 13-1500, Thermo Fisher, Rockford, IL) was used to label cells for 2 h at 37°C followed by an anti-mouse immunoglobulin G (IgG) labeled with Alexa Fluor 488 (Donkey, 2 mg/ml, 1:1000, no. A-21202, Thermo Fisher, Eugene, OR) at room temperature for 1 h. Nuclei were stained wtih 4,6-diamidino-2-phenylindole (DAPI, 10 mg/ml, 1:1000, no. D1306, Thermo Fisher, Eugene, OR) for 10 min followed by a distilled water rinse. Cells cultured in stromal medium and induced cells incubated with secondary antibody alone served as controls. Photomicrographs were obtained with a camera (DFC480, Leica Microsystems, Germany) on a fluorescent microscope (DM 4500b, Leica Microsystems).

### Transmission electron microscopy ultrastructure

Megascopic cell pellets (3 × 10^6^ cells) formed by centrifugation (260×*g*, 5 min) with cells detached as described above were fixed in 1.6% paraformaldehyde, 2.5% glutaraldehyde, and 0.03% CaCl_2_ in 0.05 M cacodylate buffer (pH 7.4) overnight. Following a 0.1-M cacodylate buffer wash, post-fixation was performed with 2% osmium tetroxide for 1 h, a distilled water wash, and incubation in 1% tannic acid for 1 h. Cell dehydration was performed with propylene oxide in an ascending ethanol series followed with Epon-Araldite (EMS, Hatfield, PA) embedding. Blocks were sectioned (80–90 nm, Ultratome Leica EM UC7, Germany) and stained with 2% uranyl acetate in maleate buffer and lead citrate for 5 min. An electron microscope (JEOL JEM 1011, Japan) with a digital camera (Hamamatsu ORCA-HR, Japan) was used for imaging.

### Transmission electron microscopy immunolabeling

Pellets of CD105^+^K14^+^ cells prepared as described above and cell constructs (described below) were fixed in 4% paraformaldehyde and 0.5% glutaraldehyde in 0.1 M phosphate buffer (PB) for 10 min. Samples were centrifuged (260×*g*, 5 min), the supernatant removed and replaced with fresh fixative, and then samples maintained on an orbital shaker (Fisher Scientific, Ottawa, Canada) at room temperature for 2 h. Following removal of the supernatant, pellets were combined with an equal volume of 3% agarose and cut into cubes following solidification. Cubes were placed into glass vials containing 0.1 M phosphate buffer, pH 7.4, washed with 0.1 M phosphate buffer and 0.08 M glycine five times for 15 min, and then washed with distilled water three times for 5 min.

Samples were dehydrated in 100% ethanol three times for 20 min each. They were then infiltrated with 100% ethanol and London resin (LR) white (1:1) for 2 h at room temperature followed by 2 h of soaking in fresh 100% LR white. Samples were embedded in 100% LR white in a beam embedding capsule and polymerized at 65°C for 24 h. Ultrathin sections (90 nm, Leica EM UC7 Microtome) were collected on a 150-mesh filmed carbon coated nickel grid (EMS FCF-150-Ni). They were blocked with 5% bovine albumin (Sigma, St. Louis, MO) in PBS for 30 min, incubated with a K15 polyclonal antibody (rabbit, 0.2 mg/ml, 1:100, Proteintech, no. 10137-1-AP, Rosemont, IL) in a buffer containing 1× phosphate-buffered saline with Tween 20 and 0.2% BSA-c (pH 7.4) for 4 h at 4°C and then washed six times with buffer, each time 5 min. Sections were incubated with a gold conjugated secondary antibody (anti-rabbit IgG, 0.15 mg/ml, 1:20, 10 nm, Cytodiagnostics, no. AC-100105, Canada) in buffer for 90 min at room temperature followed by a rinse with buffer as before. The same sections were incubated with a CD105 antibody (mouse, 0.2 mg/ml, 1:10, eBioscience, no. 12-1057-42, San Diego, CA) in buffer overnight at 4°C. Sections were washed with buffer six times for 5 min, followed by another wash in phosphate-buffered saline with Tween 20 three times, each time 5 min. They were then incubated with a gold conjugated secondary antibody (anti-mouse IgG, 0.15 mg/ml, 1:20, 20 nm, Cytodiagnostics, no. AC-200205, Canada) in buffer for 120 min at room temperature followed by six buffer rinses, 5 min each. Samples were post-fixed in 2% glutaraldehyde in PBS for 5 min. Sections were washed thoroughly in distilled water and stained with 2% uranyl acetate and lead citrate. Digital images were recorded as above.

### Stratum internum tissue decellularization

Stratum internum tissue (5 × 5 × 5 mm), harvested as described above, was soaked in 0.1% EDTA in PBS at room temperature for 1 h, then in PBS alone for another hour. Tissue was subsequently placed in 10 mM tris (hydroxymethyl) aminomethane and 0.1% EDTA in distilled water overnight at 4°C. Samples were transferred to PBS for 1 h at room temperature and then to 10 mM tris (hydroxymethyl) aminomethane with 0.5% SDS in distilled water for 24 h at 4°C. They were washed with PBS three times for 5 min and then soaked in 4 M ice-cold perchloric acid for 30 min. Samples were moved into PBS for 1 h and then lyophilized (− 55°C, VirTis BenchTop, Stanford, TX) for 12 h. Lyophilized tissues were sterilized with ethylene oxide prior to addition of cells.

### Cell construct preparation and culture

Cell pellets were prepared as described above and then resuspended in 200 μl of PBS. A total of 3 × 10^6^ cells were added to stratum internum tissue scaffolds by pipetting to give a cell density of 2.4 × 10^4^/mm^3^. Constructs were cultured in 12 well plates (Fisher Scientific, Denmark) with stromal medium for a total of 4 d under standard culture conditions (5% CO_2_, 37°C).

### Construct ultrastructure with SEM

Representative samples from each construct were fixed with 2.5% glutaraldehyde in 0.1 M sodium carcodylate buffer (pH 7.4), post-fixed in 0.1% osmium tetroxide, and dehydrated in a series of ethanol-distilled water solutions. They were sputter coated with gold and imaged with an electron microscope (FEI Quanta 200, Eindhoven, Netherlands) and digital camera (Gatan Orius SC1000A1, UK).

## Results

### Cell cytoskeleton morphology

**Figure 2.**
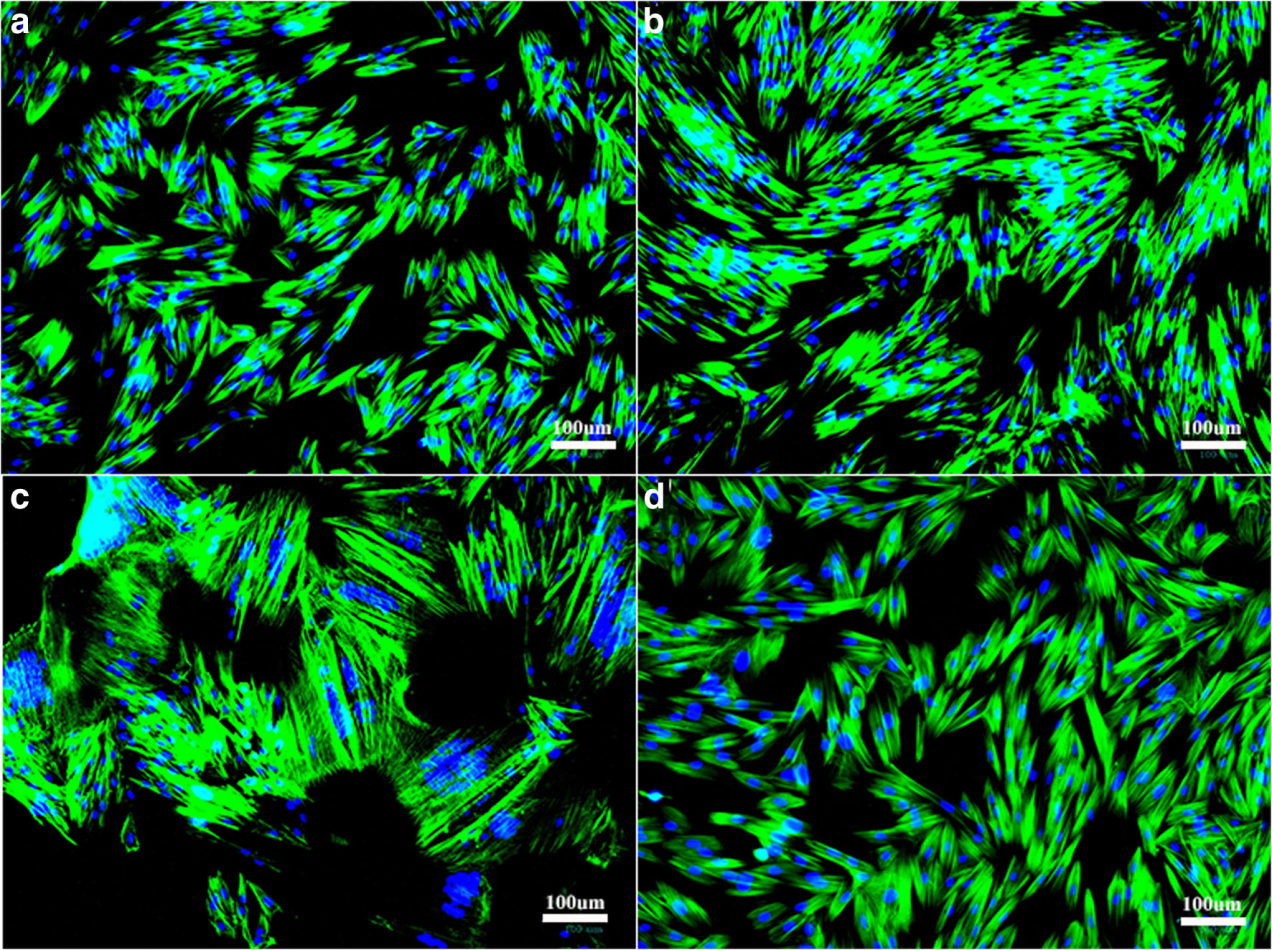
Passage 3 progenitor cells from fresh normal (**a**), inflamed (**b**), and cryopreserved normal tissue (**c**), as well as CD105^+^K14^+^ cells from fresh normal tissue (**d**) with cytoskeletal (Acti-stain™ 488 phalloidin, *green*) and nuclear (Hoechst dye, *blue*) staining.

Heterogeneous and CD105^+^K14^+^ progenitor cell isolates had typical spindle-shaped morphology with minimal cell clustering (Fig. 2). Heterogeneous cell isolates from inflamed tissue and isolates from cryopreserved, normal tissue had spindle-shaped morphology, but cells tended to aggregate in culture.

### CD105^+^K14^+^ cell multilineage differentiation (adipogenic, osteogenic, neurogenic)

**Figure 3.**
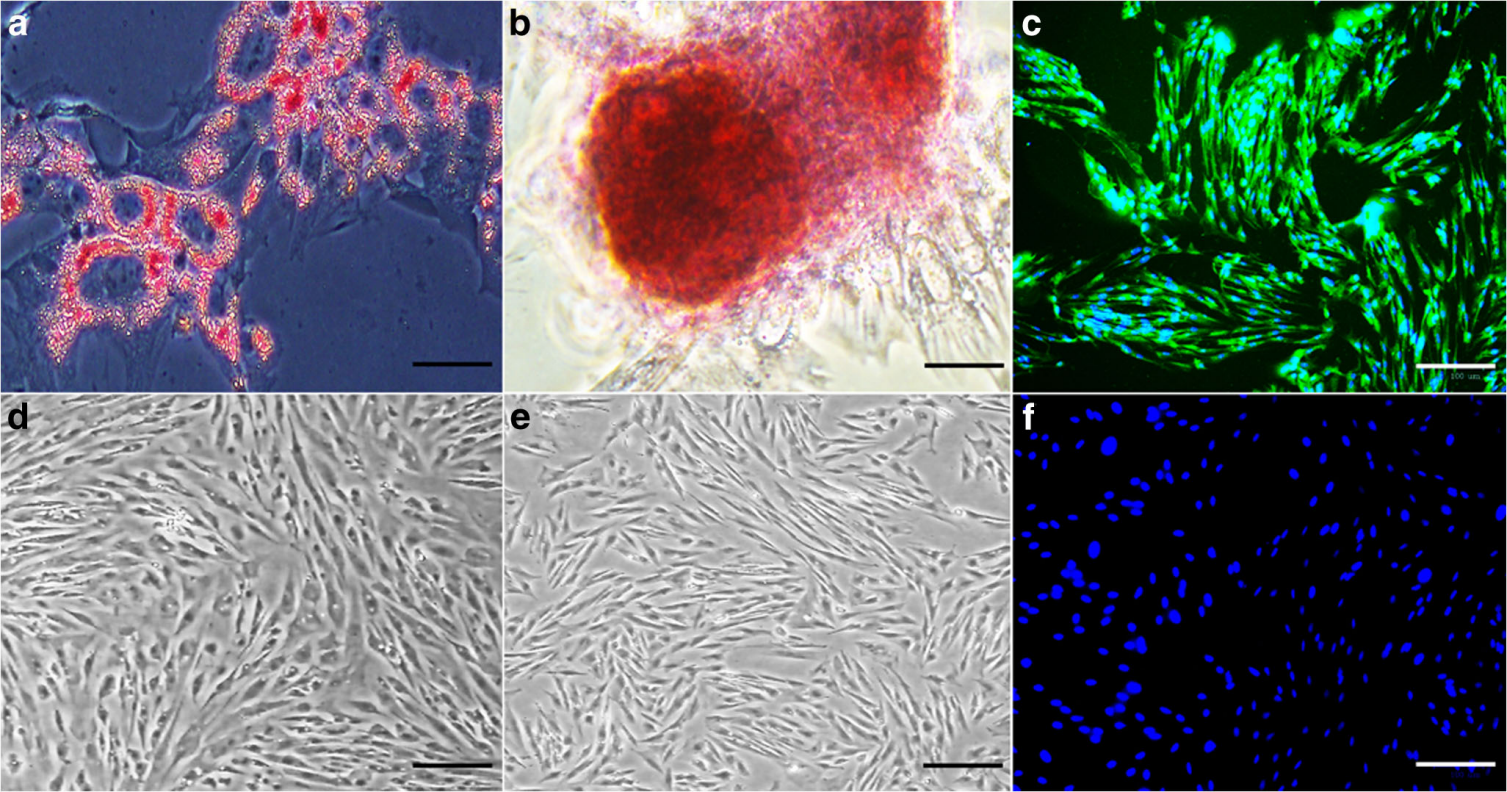
Photomicrographs of P3 CD105^+^K14^+^ cells after culture in adipogenic (**a**), osteogenic (**b**), and neurogenic (**c**) induction medium. Cells cultured in stromal medium for the same durations are also shown (**d**–**f**). Cells were stained oil red O (**a**, **d**), alizarin red (**b**, **e**), or anti-Map 2 and anti-mouse IgG-488 (green, **c**, **f**). Nuclei were stained with DAPI (blue, **c**, **f**). *Bar* = 50um (**a**, **b**); 100 μm (**c**–**f**).

Passage 3 CD105^+^K14^+^ cells showed adipocytic, osteoblastic, and neurocytic differentiation after culturing with differentiation induction medium based on histochemical and immunocytochemical staining (Fig. 3).

### Transmission electron microscopy ultrastructure

**Figure 4.**
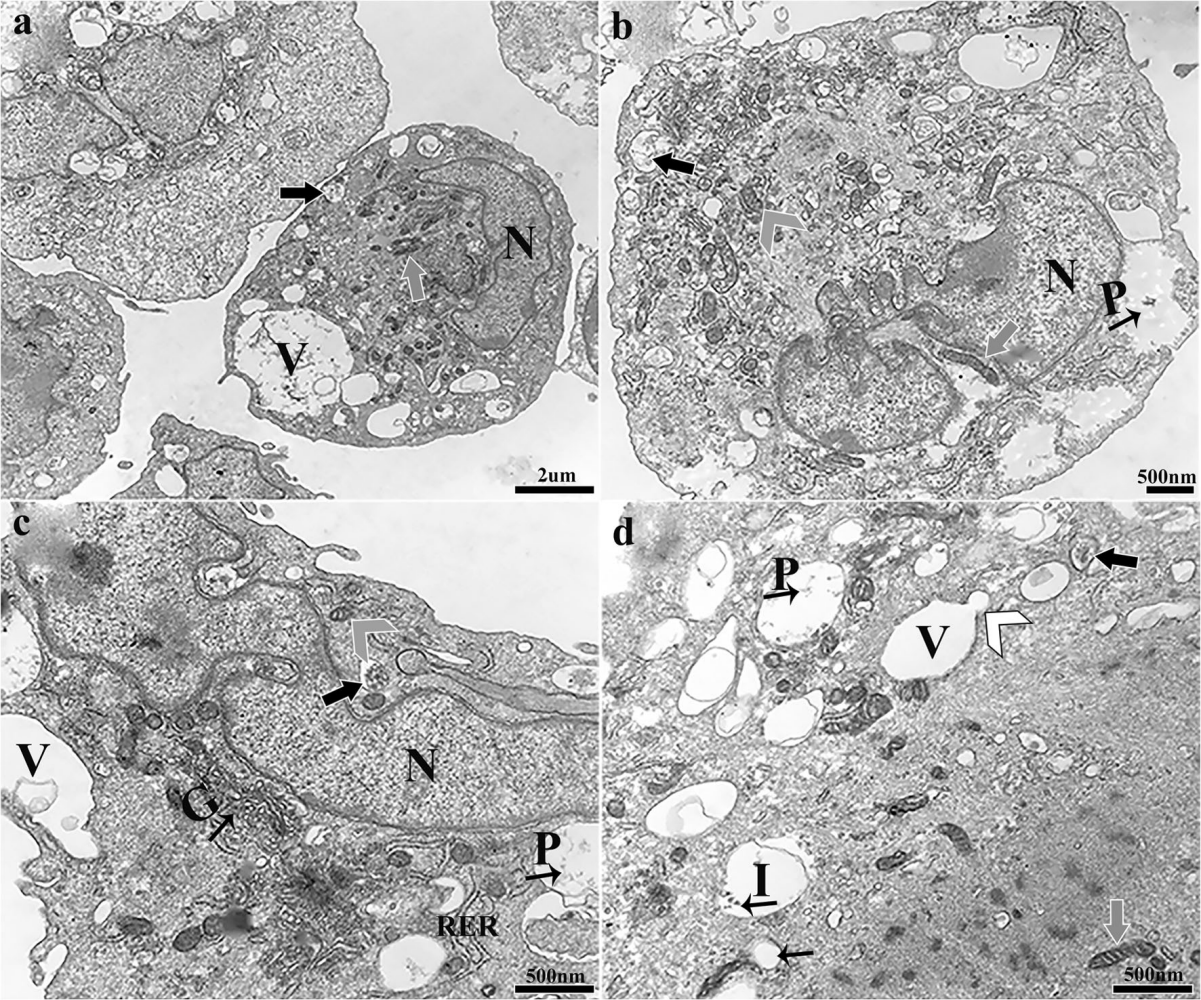
Heterogeneous progenitor cell isolates from normal hooves. Cells typically contained a large eccentric nucleus (N, **a–c**) filled with euchromatin and surrounded by golgi apparatus (*small black arrow with G*, **c**). Multiple, cytoplasmic, late (*large black arrows with white outline*, **a–d**), and early (*small black arrows*, **d**) endosomes were typically surrounded by normal (*gray arrow head, white outline*, **b, c**) and elongated (*gray arrows*, *white outline*, **a, b, d**) mitochondria and RER. Abundant large vacuoles (V, **a, c, d**) had variable contents including particles (*small black arrow with P*, **b–d**) and intraluminal vesicles (*small black arrow with I*, **d**) and were sometimes fused with early endosomes (*white arrow head*, *black outline*, **d**).

**- Fresh normal tissue.** There were distinct differences in ultrastructure among cell source environments. Progenitor cells from fresh normal tissue contained eccentric nuclei with typical euchromatin and closely approximated golgi apparatus as well as abundant, cytoplasmic endosomes that were often surrounded by mitochondria and rough endoplasmic reticulum (RER) (Fig. 4). Cell shape ranged from cuboidal to round with a relatively smooth surface. Cells contained multiple early and late endosomes, elongated mitochondria as well as large vacuoles. Early endosomes appeared as small, single compartment, membrane-bound vesicles. Late endosomes, also known as multivesicular bodies, had intraluminal vesicles and tended to be larger than early endosomes. Vacuoles tended to be large, and contents varied widely from vesicle remnants to proteinaceous and particulate material, sometimes within intraluminal vesicles and fused with early endosomes.

### Fresh inflamed tissue

**Figure 5.**
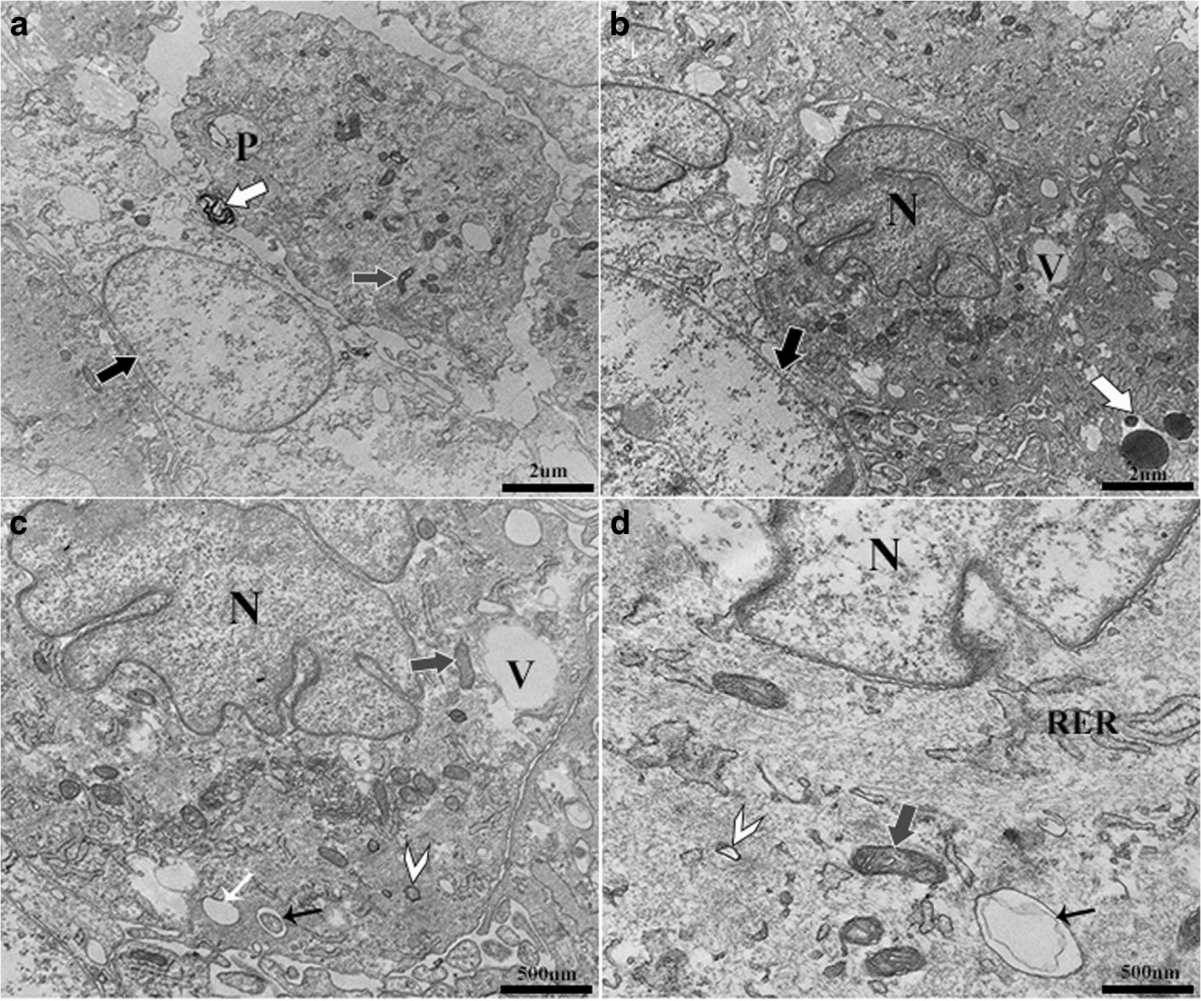
Progenitor cells from inflamed (laminitic) equine hooves. Nonviable cells (*large black arrows*, *white outline*, **a, b**) were apparent among viable cells that had an irregular surface and large nuclei (N, **b–d**) with deep membrane clefts. Cells contained lysosomes (*large white arrows*, *black outline*, **a, b**) with dark staining acidic content. Vacuoles (V, **b, c**) and early endosomes (*small white arrow*, **c**) were less prevalent than in cells from normal tissues. Endosomes were surrounded by irregular RER and mitochondria (*gray arrows*, *white outline*, **a, c, d**). Small coated vesicles (*white arrow head*, **c, d**) were often present around mitochondria. Late endosomes that often contained single, large intraluminal vesicles (*small black arrow*, **d**) were also found around early endosomes.

Cells from fresh inflamed hooves tended to be aggregated in thin sections similar to microstructural organization, and there were dead cells in various stages of degradation among viable cells (Fig. 5). The cell shape was comparable to that of cells from healthy tissue, but the surface was covered with shallow invaginations. Cells had large, irregular nuclei with large clefts in the nuclear envelope and contained lysosomes with darkly stained acidic content. Endosomes tended to be smaller and less abundant than those in cells from healthy tissue, clustered, and surrounded by mitochondria and irregular RER. Small coated vesicles were also around mitochondria. Large vacuoles were less frequent than in cells from healthy tissue. Mitochondria around vacuoles tended to be elongated.

### Cryopreserved tissue

**Figure 6.**
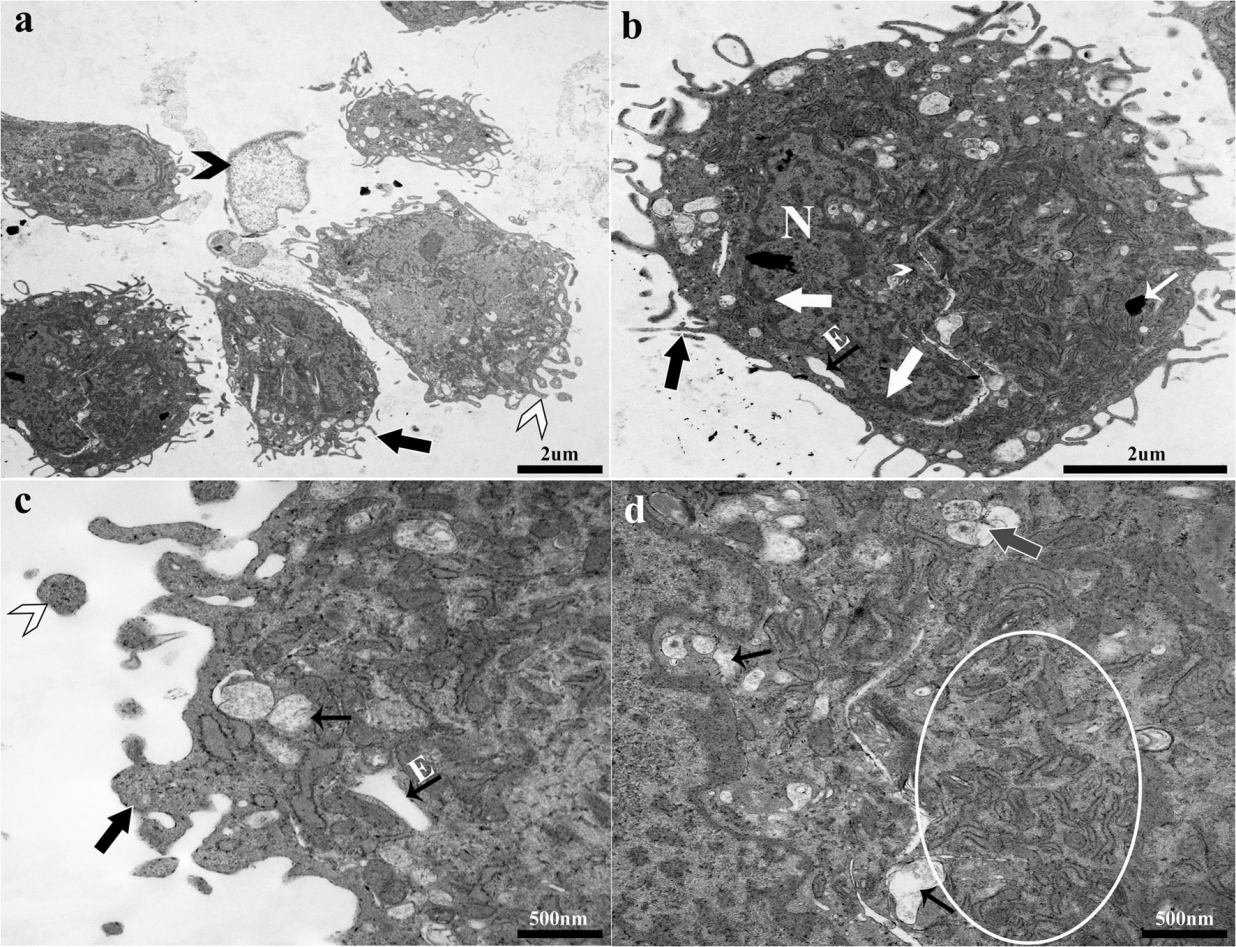
Progenitor cells from cryopreserved normal laminar tissue. The irregular cell membrane was covered by cytoplasmic protrusions and microvilli (*large black arrows*, **a–c**) that were closely associated with endocytic vesicles (*small black arrows with E*, **b, c**) and exocytic vesicles (*white arrow head*, **a, c**). Nonviable cells (*black arrow head*, **a**) were present among viable cells. Chromatin was aggregated on the nuclear envelop (*large white arrows*, **b**). Dark stained lysosomes (*small white arrow*, **b**), small vacuoles (*small black arrows*, **c, d**) containing proteinaceous material, and multivesicular bodies were present (*gray arrow*, *white outline*, **d**) and surrounded by abundant, poorly organized RER (*white circle*, **d**).

Progenitor cells from cryopreserved normal tissue were characterized by dense, exocytic vesicles, and an irregular cell membrane covered with cytoplasmic protrusions, microvilli on the outer surface, and endocytic vesicles on the inner surface (Fig. 6). The cell cytoplasm was electron-dense and amorphous. Cell organelles consisted primarily of poorly organized RER, lysosomes with darkly stained material, small vacuoles with proteinaceous material, and endocytic vesicles. Large nuclei contained aggregated chromatin close to the nuclear membrane. Nonviable cells in various stages of degradation were present among viable cells.

### CD105^+^K14^+^ cells

**Figure 7.**
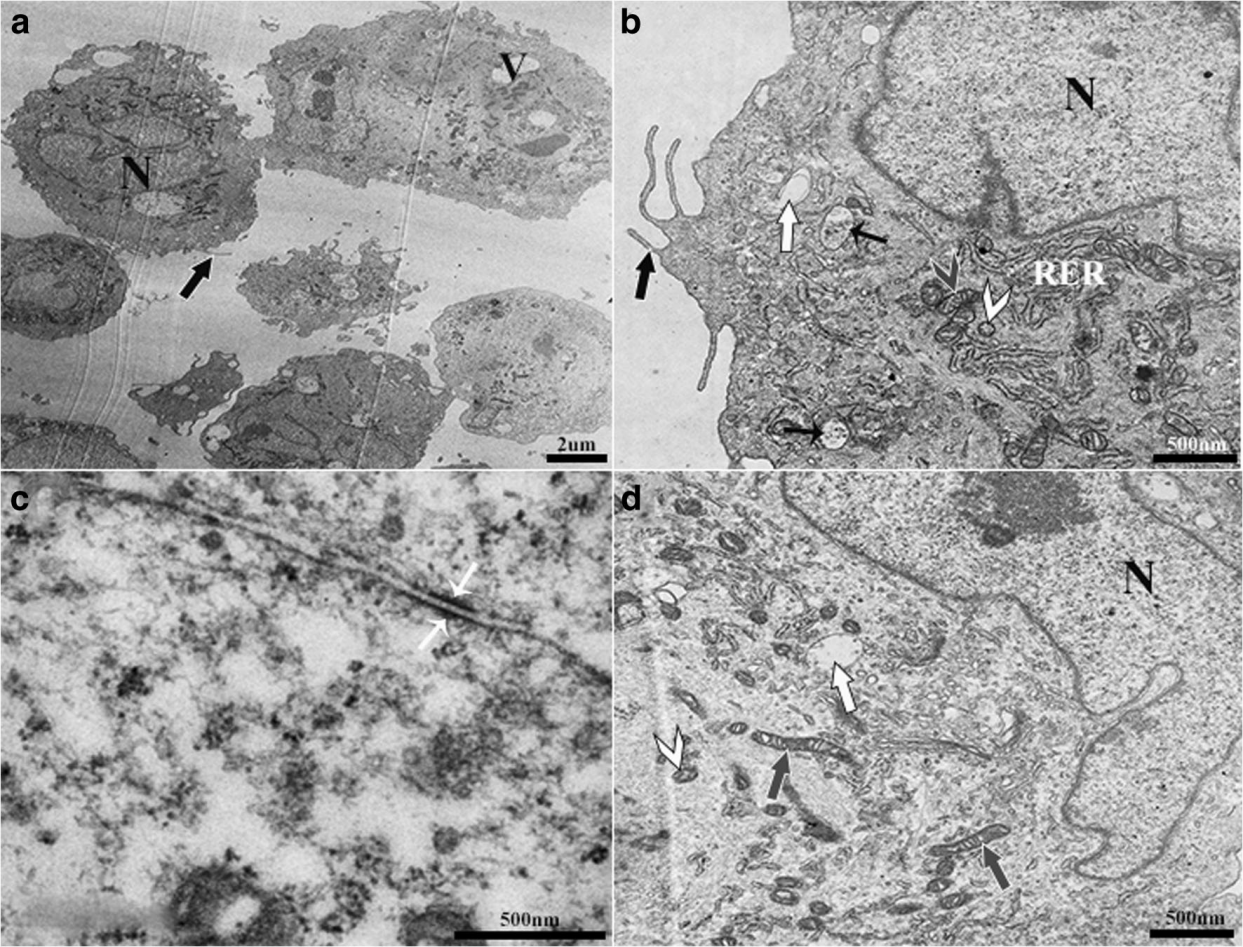
Double positive (CD105^+^K14^+^) progenitor cells from normal equine hoof tissue. Cells had a large nucleus (N, **a, b, d**), infrequent microvilli (*large black arrow*, *white outline*, **a, b**) on the surface, and sparse, large vacuoles (V, **a**). Multivesicular bodies (*small black arrows*, **b**) and endocytic vesicles (*large white arrows*, *black outline*, **b, d**) were present among mitochondria (*gray arrow head*, **b**) and RER. Desmosomes were present (*small white arrows*, **c**) between cells. Elongated mitochondria (*gray arrows*, **d**) and nonelongated mitochondria (*gray arrow head*, **b**) close to the nucleus were surrounded by small coated vesicles (*white arrow heads*, **b, d**).

Double positive cells from normal fresh tissue were typically cuboidal and had infrequent microvilli on the cell surface (Fig. 7). Large nuclei had prominent nucleoli and euchromatin, and the ratio of nucleus to cytoplasm tended to be large. Endosomes and vesicles were infrequent, but those present were surrounded by mitochondria and RER. Desmosomes were frequently present between cells. Cells contained multivesicular bodies as well as small coated vesicles from early endosomes around RER and mitochondria. Some mitochondria close to the nucleus had an elongated morphology.

### Transmission electron microscopy immunolabeling (K15, CD105)

**Figure 8.**
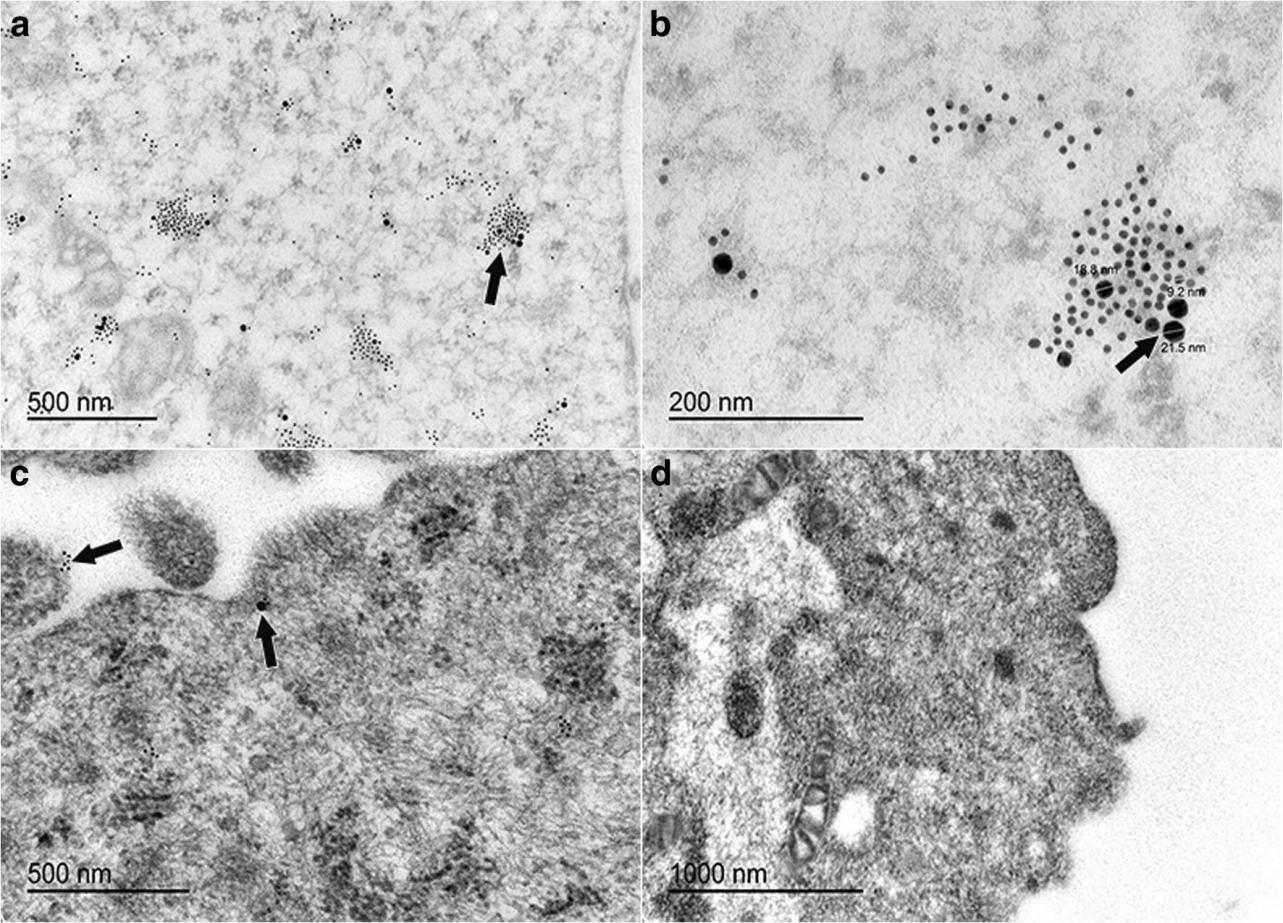
Gold particle-labeled secondary antibodies against K15 (10 nm) and CD105 (20 nm) in equine laminar progenitor cells from normal hooves sorted by co-expression of K14 and CD105 antigens. Gold labels (*black arrows*, **a–c**) localized throughout the cell cytoplasm (**a**, **b**) and in close proximity to the cell membrane (**c**), both singly and together. There was no labeling of cells treated only with gold particle-labeled secondary antibodies (**d**).

Gold particle–labeled secondary antibodies to K15 (10 nm) and CD105 (15 nm) localized singly or together in the cytoplasm of sorted cells (CD105^+^K14^+^) and sometimes in close proximity to the cell membrane (Fig. 8a–c). There was no labeling in cells exposed to secondary antibodies alone (Fig. 8d).

### Construct ultrastructure

**Figure 9.**
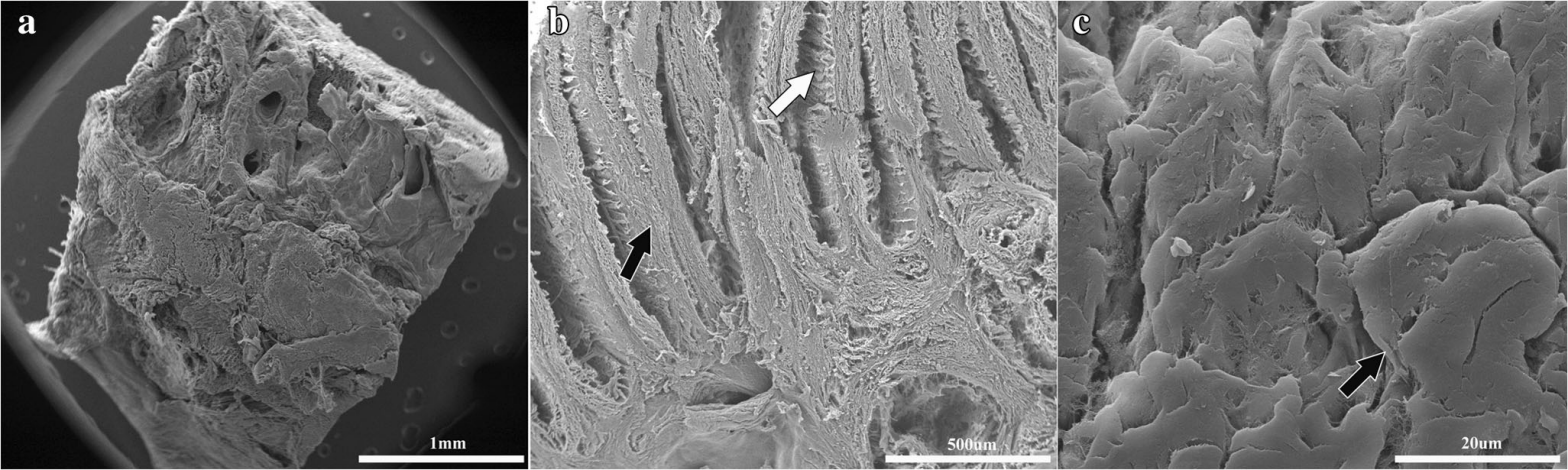
Decellularized hoof stratum internum (**a–c**). The primary (*black arrow*, **b**) and secondary (*white arrows*, **b**, **c**) epidermal lamellae architecture was retained during the decellularization process, but it was devoid of extracellular matrix and cells.

**- Tissue scanning electron microscopy.** The primary and secondary epidermal lamellae structures were retained following decellularization, but they were devoid of extracellular matrix and cells (Fig. 9).

### Cell and tissue construct scanning electron microscopy

**Figure 10.**
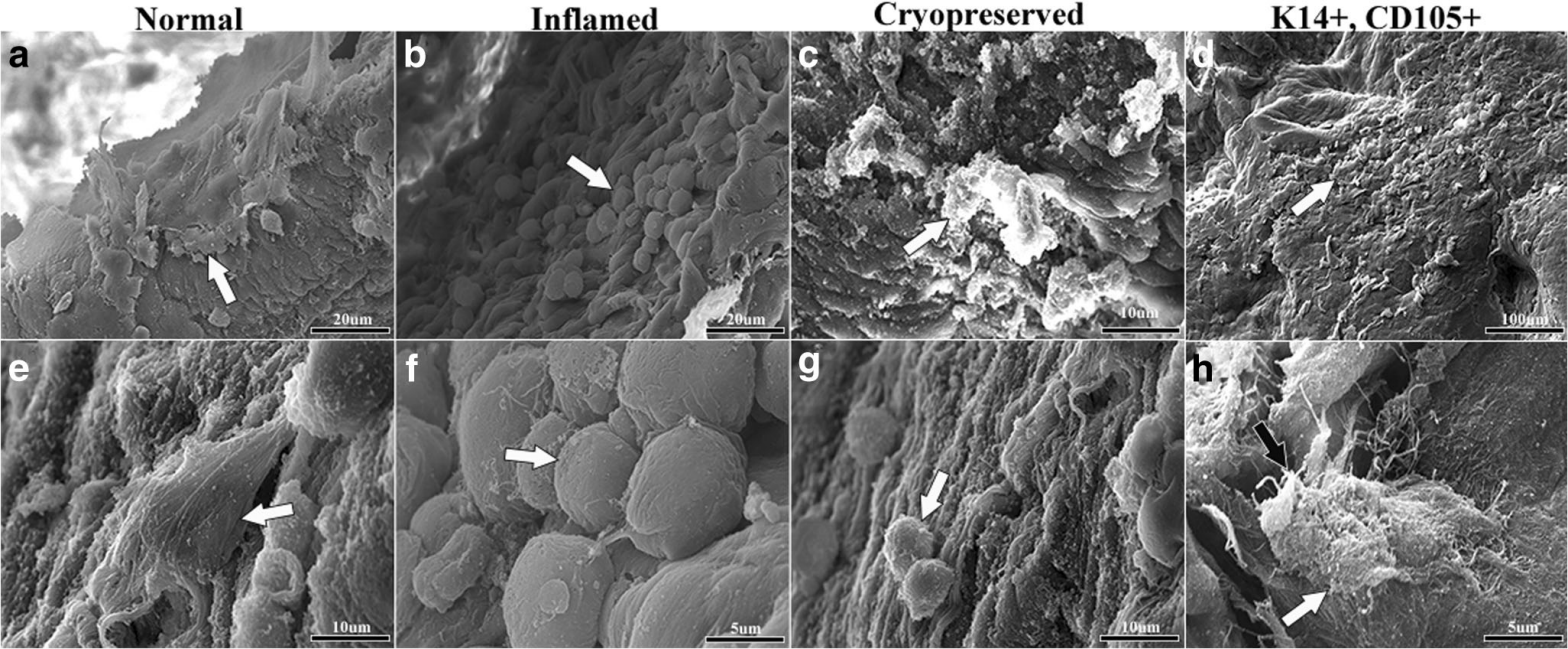
Scanning electrophotomicrographs of equine hoof progenitor cells on decellularized stratum internum tissue scaffolds after 4 d of static culture in stromal medium. Progenitor cells from normal tissue were strongly attached to the scaffold (*white arrows*, **a**, **e**). Progenitor cells from inflamed tissue had a round shape and were present in clusters (*white arrows*, **b**, **f**). Cells from cryopreserved tissue appeared in scarce clusters and were round (*white arrows*, **c**, **g**). Double positive cells (CD105^+^K14^+^) were well attached to the tissue scaffold (*white arrows*, **d**, **h**) and surrounded by fibrous extracellular matrix (*black arrow*, **h**).

Cultured cells from fresh normal and laminitic tissue, from cryopreserved normal tissue, and CD105^+^K14^+^ cells from fresh normal tissue attached to decellularized scaffold after 4 d of static culture in stromal medium (Fig. 10). There were abundant cells in all samples except those from cryopreserved tissues which were present in scarce clusters. The cells from laminitic tissue were also arranged in clusters, and cells from both had a round shape. Neither had detectable extracellular matrix around the cells. In contrast, cells from normal tissue were flattened and tightly adhered to the scaffold surface, and double positive cells were well adhered and embedded in early, fibrous extracellular matrix.

### CD105^+^K14^+^ cell and tissue construct transmission electron microscopy

**Figure 11.**
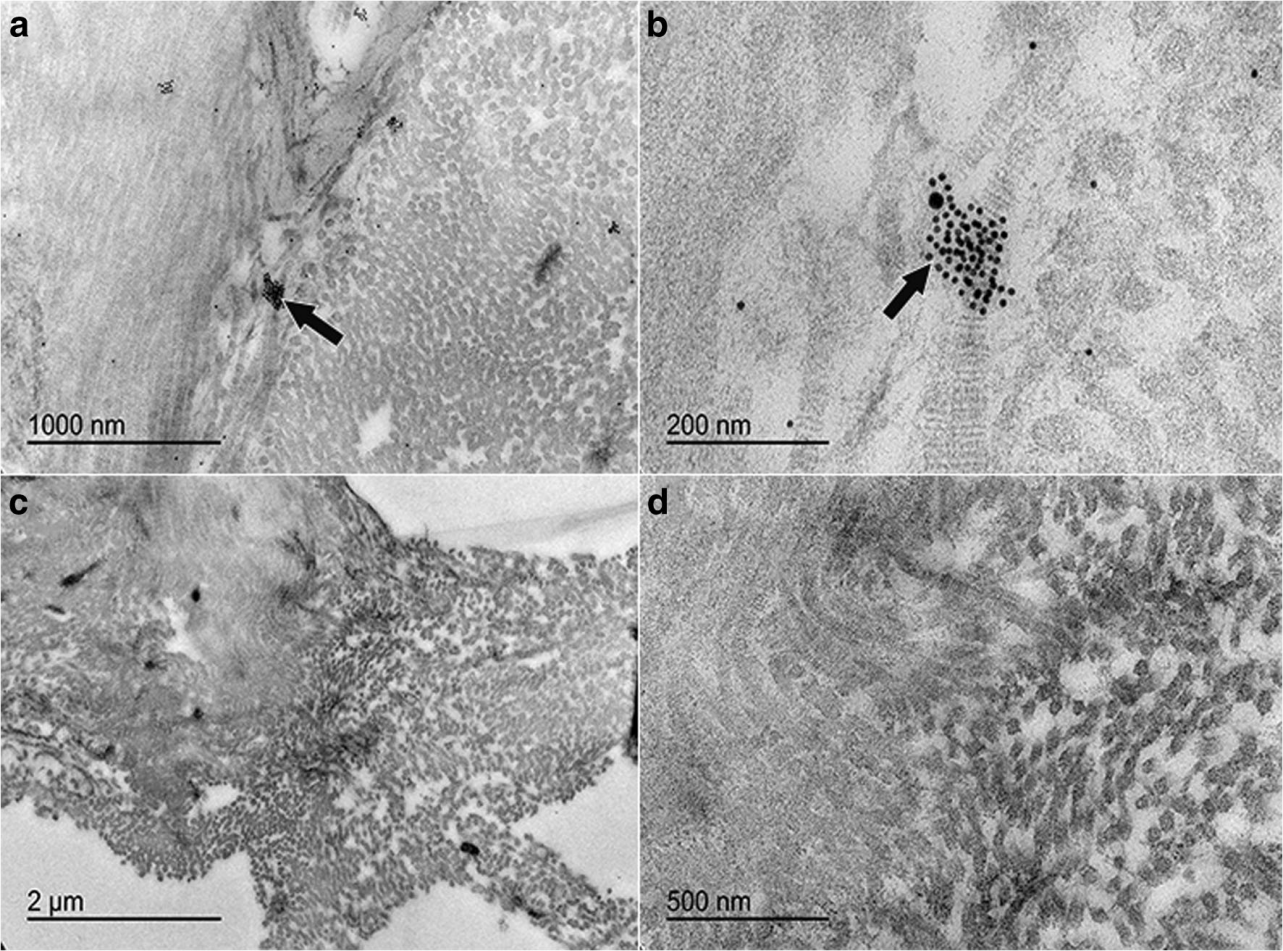
Construct sections demonstrating labeling of K15 (10 nm) and CD105 (20 nm) protein antigens in decellularized epidermal stratum interim following 4 d of culture with CD105^+^K14^+^ hoof progenitor cells in stromal medium (*black arrows*, **a**, **b**). There was no labeling of decellularized tissue without cells (**c**, **d**).

Cell constructs with double positive sorted cells (CD105^+^K14^+^) contained ectodermal and mesodermal protein antigens after 4 d of culture based on localization of gold labeled secondary antibodies against K15 (10 nm) and CD105 (20 nm) in construct sections both together and separately (Fig. 11).

## Discussion

The results of this study clearly demonstrate important distinctions in progenitor cell morphology among normal, inflamed and cryopreserved equine hoof tissues and confirm unique structural characteristics of progenitor cells with both mesodermal and ectodermal antigens. This vital morphological information is invaluable to understanding the behavior and predicting the potential of adult progenitor cells harvested from the unique dermal-epidermal interface in the equine hoof that shares many features with a similar tissue interface in the human fingernail (Clement et al. 2013; Momin et al. 2014). Differences among progenitor cells provide important clues about tissue formation and maintenance by the cells in vivo, the de novo tissue forming potential of the cells in vitro, and the impact of cryopreservation and antigen-based sorting on the cells. The results of this study support that differences are maintained over multiple passages under optimal culture conditions and on tissue-based scaffolds. They also confirm that harvest tissue condition as well as post-harvest preservation and cell selection all impact cell structure, a general reflection of function (Li and Xie 2005; Bernstein et al. 2006).

The gross morphology of primary cell isolates was differentiated only by the tendency of cells from inflamed and cryopreserved tissue to form clusters. The spindle shape is typical of heterogeneous equine cell isolates from either mesodermal or ectodermal tissues, including equine adipose tissue and bone marrow derived multipotent stromal cells (MSCs), epidermis-like stem cells, and skin fibroblasts (Vidal and Lopez 2011; Broeckx et al. 2014; Alkhilaiwi et al. 2018). The tendency to form clusters can be associated with cell maturity and inflammation (Lotz et al. 2010). Previous reports indicate that cryopreservation tends to “age” cells, and inflammation may have a similar effect (Hoshiyama et al. 2015; Duan et al. 2018). The ultrastructural cubiodal or round shapes of the progenitor cells in this study are typical of immature cells from ectoderm in multiple species including the rat, and human MSCs assume a polygonal morphology after epidermal induction (Chepko and Dickson 2003; Jin et al. 2011). The large ratio of nucleus to cytoplasm apparent in CD105^+^K14^+^ cells is a sign of early-stage progenitor cells (Akiyama et al. 2000). Overall, the cells share similarities with epidermal stem cells and MSCs. Additional work with various immunophenotypes will be required to determine the embryonic origin of equine hoof progenitor cells.

The morphology of cells from fresh normal tissue, sorted (CD105^+^K14^+^) and unsorted, was largely distinguishable by the presence of microvilli, fewer and smaller vacuoles, and more late endosomes dispersed throughout the cytoplasm in sorted cells. The presence of microvilli on the sorted cells may indicate greater commitment to intense exocytic and endocytic activity characteristic of higher plasticity (Sauvanet et al. 2015). Late endosomes are a sign of a high capacity for intracellular protein recycling required for successful differentiation in umbilical cord blood-derived multilineage progenitor cells (Berger et al. 2006). Numerous vacuoles, thought to originate from dilated endoplasmic reticulum, in the heterogeneous cell isolates also indicate cytoplasmic endo- and exocytotic activities as documented in equine progenitor cells from other tissues (Pasquinelli et al. 2007; Pascucci et al. 2010). In both, the ultrastructural appearance was consistent with healthy progenitor cells from equine hoof tissue.

Cell sorting facilitates isolation of homogeneous cell populations that are reported to have more predictable in vivo and in vitro behavior (Kollet et al. 2002; Emara et al. 2017). The CD105^+^K14^+^ subset of the heterogeneous progenitor cells from normal hooves may have greater capacity for differentiation into either ectodermal or mesodermal tissue (Gu et al. 1994). This is suggested by the presence of filopodia-like microvilli and cell differentiation into mesodermal and ectodermal lineages that are both characteristics of cells capable of epidermal to mesodermal transition (EMT). An EMT cell signaling pathway, Wnt, has been identified in hoof tissue (Kalluri and Weinberg 2009; Wang et al. 2013b). Notably, immunostaining further confirmed that the cells continued to express antigens from both tissue lineages under standard culture conditions designed to maintain a progenitor cell morphology in this study. Hence, the observed morphology is maintained in culture. Future work is needed to confirm the superior plasticity and EMT potential of the sorted cells.

The cells from cryopreserved tissue showed features consistent with apoptosis including degranulation of prevalent RER, aggregated chromatin, and an irregular cell membrane with abundant exocytic vesicles (Campello and Scorrano 2010; He et al. 2018). These findings are consistent with established knowledge that cells are somewhat “aged” by thermally induced stasis and show signs of necrosis, as well as a lower isolation rate, lower colony recovery rate, and lower reprogramming efficiency than those from fresh tissue (Newton et al. 1999; Seo et al. 2005; Sproul et al. 2014; Canton et al. 2016). Thus, though the cells were capable of some proliferation and passage, they were unlikely to have much de novo tissue forming capacity. Notably, the cells were maintained at a low temperature consistent with short-term preservation versus ultralow temperature for long-term storage. As such, the results are specific to the conditions tested. It is possible that customization of cryopreservation techniques for protection of cells within tissues, isolation, and preservation of cells separately from tissue, or maintenance of the cells in the vapor phase of liquid nitrogen may have less or different effects on the cells.

Another important finding in this study was differences in progenitor cells from normal and inflamed tissues. The presence of lysosomes and degraded dead cells among cell isolates from inflamed tissue suggests that the cells were functioning more in tissue breakdown than production and maintenance (Kroemer and Jaattela 2005). The presence of cytokines, chemokines, and matrix metalloproteinases in inflamed hooves likely influences progenitor cell behavior and reduces plasticity of the cells (Jaiswal et al. 2000; Liao et al. 2011; van Eps et al. 2012), in part due to direct damage to the cells themselves (Elner et al. 1990; Prasanna et al. 2010). It is also thought that progenitor cells assume the characteristics of their environment (Kopen et al. 1999). This finding is especially important in light of the fact that the cells had some proinflammatory or catabolic morphology including lysosomes, irregular RER, and few cytoplasmic vacuoles, though they were continuously cultured under optimum conditions following harvest. This is in contrast to some reports that cells recover in an ideal environment (Chen et al. 2014). Targeted work is necessary to align actual cell behavior with the morphologies observed in this study.

Many of the features of 2D culture observed in this investigation were maintained in 3D culture, validating the 2D findings. It is especially important to note that, while cells from the heterogeneous and sorted cohorts attached to the scaffold, only the CD105^+^K14^+^ cells produced detectable ECM in a relatively short period of time. This further confirms the greater plasticity and neotissue forming capacity of the sorted cell population conveyed by the morphology. Cells from inflamed and cryopreserved tissue did not attach well, consistent with the catabolic and apoptotic appearance, respectively, observed in 2D. Decellularized tissue scaffolds have advantages over synthetic biomimetic scaffolds, and they may be the best option for the unique equine hoof tissue interface (Shah et al. 2014; Mazza et al. 2015). Notably, the CD105^+^K14^+^ cells produced ectodermal and mesodermal proteins on scaffold constructs. This is promising for translation to clinical application because implantation of progenitor cell-scaffold constructs with well-integrated, functional cells has the best potential for normal tissue formation in vivo (Sundelacruz and Kaplan 2009; Rustad et al. 2012).

This study is limited by a single focus on cell ultrastructure. Prediction of cell behavior based on ultrastructural morphology is well documented (Klymkowsky et al. 1983; Strunov et al. 2016). Additionally, the cell behaviors in 2D culture were recapitulated in a 3D environment. As noted above, additional side-by-side comparisons of cell behaviors are required. Nonetheless, this novel work establishes a reproducible baseline on which to design future studies surrounding equine hoof progenitor cells.

## Conclusions

A major attribute of this study is the direct comparison of the ultrastructure of progenitor cells from multiple pre- and post-harvest conditions that clearly demonstrates important differences among them. Taken together, the new information reveals that progenitor cells from the equine hoof stratum internum have characteristics of undifferentiated cells with variable metabolic and synthetic capabilities determined by preharvest tissue conditions and post-harvest cryopreservation or sorting. The ultrastructural morphology information in this study contributes to understanding of equine hoof progenitor cells to predict their potential contributions to tissue maintenance, healing, and damage as well post-implantation behavior.
